# Flower-Like MoSe_2_/MoO_2_ Composite with High Capacity and Long-Term Stability for Lithium-Ion Battery

**DOI:** 10.3390/nano9091256

**Published:** 2019-09-05

**Authors:** Qiuyan Hao, Guoliang Cui, Yan Zhao, Zhumabay Bakenov

**Affiliations:** 1 School of Materials Science and Engineering, Hebei University of Technology, Tianjin 300130, China; 2 Institute of Batteries LLC, National Laboratory Astana, Nazarbayev University, 53 Kabanbay Batyr Avenue, Nur-Sultan 010000, Kazakhstan

**Keywords:** MoSe_2_/MoO_2_ composite, anode, LIBs, capacity, long-term cycle performance

## Abstract

A simple method is developed for the preparation of MoSe_2_/MoO_2_ composite with a flower-like structure for high-performance lithium-ion batteries (LIBs). MoSe_2_ could lead to fast and facile movement of Li^+^ due to its larger interlayer spacing. Meanwhile, MoO_2_ could protect the lamellar structure of MoSe_2_ from being destroyed in the charging/discharging processes to maintain the required active surface to electrolytes. In addition, the flower-like structure of the composite could effectively alleviate the volume expansion during charging/discharging. As LIBs are anode material, MoSe_2_/MoO_2_ composite demonstrates an excellent specific discharge capacity of 1042 mAh g^−1^ after 100 cycles at 0.1 A g^−1^, which is attributed to the synergistic effects of MoSe_2_ and MoO_2_ in the composite.

## 1. Introduction

Lithium-ion batteries (LIBs) have been commercialized successfully in the past few decades and have made considerable progress in various fields, such as portable electronic products and energy storage devices for electric/hybrid vehicles [1,2,3,4,5]. Nonetheless, the commercial graphite anode is subjected to low specific capacity of 372 mAh g^−1^ and a slow insertion/extraction kinetics of Li^+^, which seriously restricts the further application of LIBs in the development of electrical fields [6,7,8]. Up to now, the theoretical specific capacities of transition metal dihalogenated hydrocarbons and oxides are higher than carbon-based anodes, so they have been researched extensively as anode materials for LIBs [9,10,11,12,13,14]. Cao and coworkers reported a nanocomposite with Molybdenum dioxide (MoO_2_) nanoparticles embedded into layered Molybdenum selenide (MoSe_2_), delivering a reversible capacity of 520 mAh g^−1^ at 2 A g^−1^ after 400 cycles [15]. However, the preparation process of this anode is relatively complex.

MoSe_2_ is a typical transition metal dichalcogenide, with a lamellar structure, which is similar to MoS_2_ [16,17,18]. The layer spacing of MoSe_2_ is 0.64 nm, which is larger than graphite (0.34 nm) and MoS_2_ (0.62 nm) [19,20]. The large interlayer spacing of MoSe_2_ favors the Li^+^ intercalation/deintercalation process, but the large volume expansion leads to poor cyclic performance [21,22,23,24,25]. As a typical transition metal oxide, MoO_2_ could also accomplish the Li^+^ insertion/extraction process [26,27]. Meanwhile, MoO_2_ possesses merits of low resistivity, high chemical stability, and electrochemical activity, so it has been used widely in the research of LIB anode materials [28,29,30].

Herein, we introduce a simple way of synthesizing a MoSe_2_/MoO_2_ composite as an advanced anode material. It is validated that the flower-like MoSe_2_/MoO_2_ composite exhibits excellent electrochemical performance, attributing to the synergy of the homogeneous nanoscale composite. The combination of high specific capacity MoSe_2_ and high electrochemically active MoO_2_ make the composite exhibit great potential for LIBs.

## 2. Materials and Methods

### 2.1. Material Preparation

During the standard procedure, 2.0 mM Se powder and 0.8 mM Na_2_MoO_4_ were dissolved in 5 mL hydrazine hydrate and 15 mL deionized water, respectively, then two solutions were mixed and stirred evenly. The hydrothermal reaction was performed for the mixed solution at 160 °C for 24 h. After the reaction, the precipitate was washed alternately with ethanol and deionized water, and dried at 60 °C for 12 h. Then the temperature of the tube furnace was raised to 300 °C, the as-prepared MoSe_2_ was calcinated for 5 min in a tube furnace in air atmosphere, and cooled to room temperature in the Ar atmosphere.

### 2.2. Characterization

The crystalline structure was tested using the X-ray diffractometer (XRD, Rigaku3014) with Cu-Kα1 radiation (λ = 0.15418 nm). The morphologies of the samples were observed by using a Nova NanoSEM 230 and a JEOL JEM-2100 TEM. The chemical composition of the samples was investigated by using X-ray photoelectron spectroscopy (XPS, Thermo ESCALAB 250) with an Al-Kα radiation. The Brunauer–Emmett–Teller (BET) surface area was tested by V-Sorb 2800P in an N_2_ atmosphere.

### 2.3. Electrochemical Measurements

The electrochemical measurements were evaluated using CR-2032 coin cells. The active materials were mixed with conductive carbon and polyvinylidene fluoride (PVDF) at a weight ratio of 7:2:1 to prepare the electrodes. The resultant slurry wasspread on copper foil and dried in vacuum at 60 °C for 12 h, and the average mass loading of each electrode was about 2.4 mg cm^−2^. CR2032 cells were assembled with lithium foil, electrolyte, and Celgard 2400 separator in an argon-filled glove box. The non-aqueous electrolyte consisted of 1 M lithium hexafluorophosphate (LiPF_6_) dissolved in a mixed solution of ethylene carbonate/dimethyl carbonate (EC/DMC, 1:1 in volume). Galvanostatic charge-discharge measurements were performed on a CT2001A test system with a voltage range of 0.01–3 V. Cyclic voltammetry (CV) curves were tested on CHI605C electrochemical workstation (0.01–3 V; 0.1 mV s^−1^). Electrochemical impedance spectroscopy (EIS) was executed at a frequency range of 0.01 Hz to 100 kHz on CHI660c electrochemical workstation.

## 3. Results and Discussion

**Figure 1:**
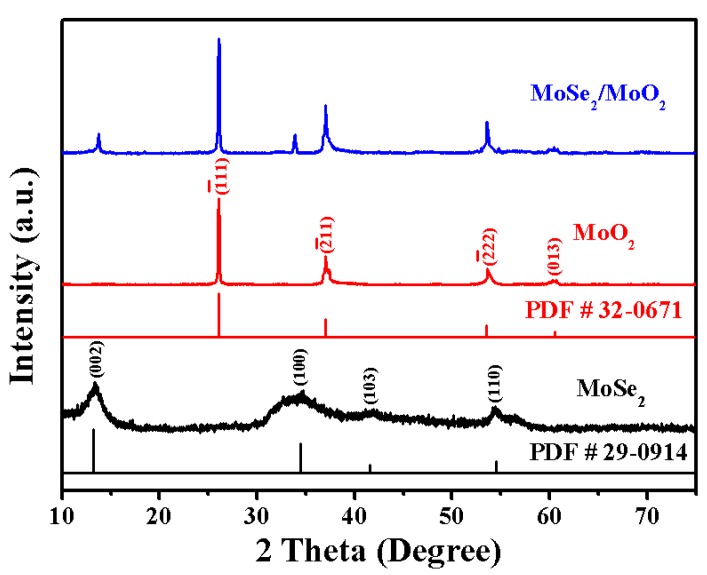
XRD pattern of MoSe_2_, MoO_2_, and the MoSe_2_/MoO_2_ composite.

The structural properties of the MoSe_2_/MoO_2_ composite are investigated by XRD in the 2θ range from 10 to 75°. Figure 1 shows the XRD patterns of the MoSe_2_/MoO_2_ composite, compared with the relevant PDF **#** 29-0914 and PDF **#** 32-0671, the hexagonal MoSe_2_ phase with a space group of P63/mmc, and the monoclinic MoO_2_ phase with a space group of P21/n are matched well with the PDF cards, respectively. The peaks at 2θ = 13.3°, 34.4°, 41.5°, 54.4° are ascribed to the (002), (100), (103), (110) planes of MoSe_2_, respectively. The other three peaks at 26.0°, 37.1°, 53.5°, and 60,5°, are assigned to (11), (11), (22), and (013) planes of MoO_2_, respectively. The crystallite sizes of the original MoSe_2_ are calculated using the Scherrer formula to be 1.7 nm and increased to 3.8 nm after calcination. Consequently, the diffraction peaks of MoSe_2_ in the MoSe_2_/MoO_2_ composite becomes sharp. These results suggest that the MoSe_2_/MoO_2_ composite has been successfully synthesized.

**Figure 2:**
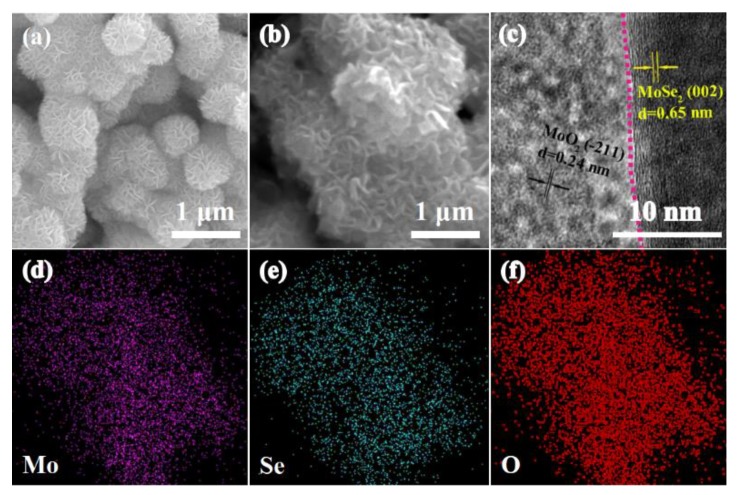
(**a**,**b**) SEM images of MoSe_2_ and MoSe_2_/MoO_2_ composite; (**c**) HRTEM image of MoSe_2_/MoO_2_ composite; (**d**–**f**) EDX mapping of MoSe_2_/MoO_2_ composite.

Figure 2a shows the scanning electron microscopy (SEM) image of MoSe_2_; it can be clearly observed that MoSe_2_ exhibits a flower-like structure formed by the self-assembly of a lamellar structure. A crumpled feature of the MoSe_2_/MoO_2_ composite could be observed in Figure 2b, which is caused by the change of morphology after calcination in air atmosphere. Figure 2c shows the high resolution transmission electron microscope (HRTEM) image of the MoSe_2_/MoO_2_ composite. The lattice fringes with 0.65 and 0.24 nm spacing could be observed in the MoSe_2_/MoO_2_ composite, which corresponds to the (002) plane of MoSe_2_ and (11) plane of MoO_2_, respectively. Meanwhile, we could observe that there is a obvious interface between MoSe_2_ and MoO_2_ marked with pink dotted lines. EDX mapping of Mo, Se, and O elements are displayed in Figure 2d–f, indicating uniform distributions of Mo, Se, and O components over the MoSe_2_/MoO_2_ composite.

**Figure 3:**
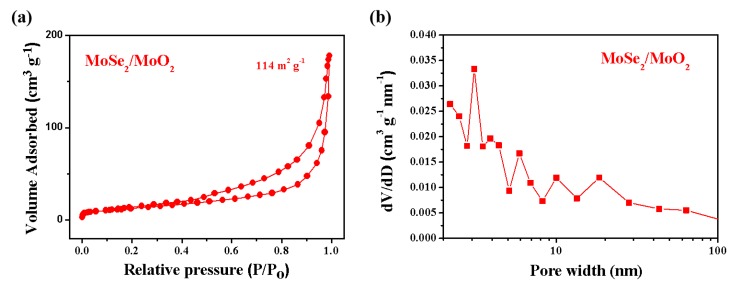
(**a**) N_2_ adsorption/desorption isotherms of the MoSe_2_/MoO_2_ composite; (**b**) Pore-size distribution of the MoSe_2_/MoO_2_ composite.

The structural properties of MoSe_2_/MoO_2_ composite are determined by N_2_ adsorption measurement. As shown in Figure 3a, the as-prepared MoSe_2_/MoO_2_ composite has a large surface area of 114 m^2^ g^−1^. The pore size distribution curve displays a narrow pore distribution of 2–4 nm, indicating there are abundant micropores and few mesoporous structures (Figure 3b). All the results show that the MoSe_2_/MoO_2_ composite has a high specific surface area and porosity; this is beneficial for the infiltration of the electrolyte to electrode enhancing the charge transfer during the process of Li^+^ insertion/extraction and adapting the huge volume change.

**Figure 4:**
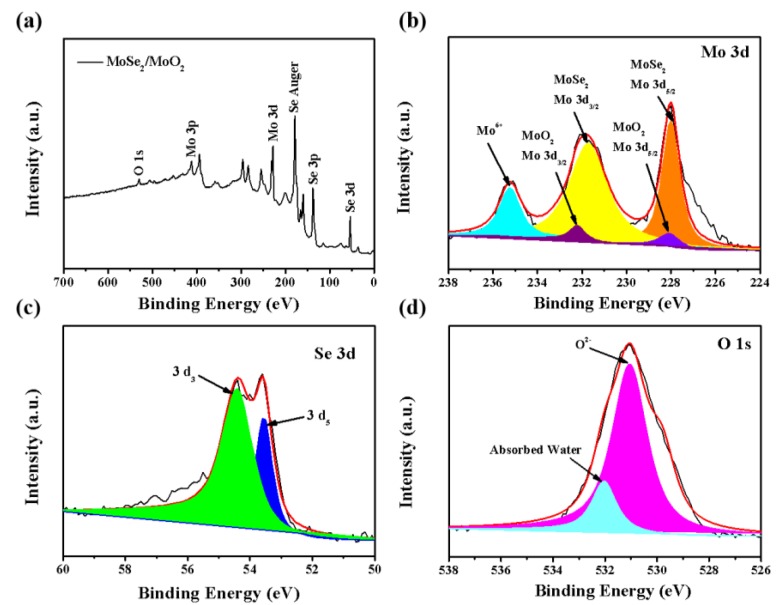
XPS spectra of the MoSe_2_/MoO_2_ composite: (**a**) a survey spectra; (**b**) Mo 3d; (**c**) Se 3d; (**d**) O 1s core level spectra.

The composition of the MoSe_2_/MoO_2_ composite is further characterized using X-ray photoelectron spectroscopy (XPS). Figure 4a shows the XPS survey spectrum of the MoSe_2_/MoO_2_ composite. The Mo 3d spectrum (Figure 4b) shows that the peaks at 231.7 eV for Mo 3d_3/2_ and 227.9 eV for Mo 3d_5/2_ are assigned to the Mo-Se bond in the MoSe_2_ [31]. The other two peaks at 232.2 and 228.1 eV are attributed to the Mo 3d_3/2_ and Mo 3d_5/2_ of Mo^4+^ in the MoO_2_, respectively [32]. Additionally, the peak at 235.2 eV corresponding to Mo 3d_3/2_ of Mo^6+^ can be ascribed to the slight oxidation of MoO_2_ by calcination in the air atmosphere [33,34]. The Se XPS spectrum (Figure 4c) shows two peaks at 54.4 eV and 53.6 eV, corresponding to Se^2-^ in MoSe_2_ [31]. The O 1s XPS peak (Figure 4d) corresponds to MoO_2_ in the Mo(IV)-O bond at 531.1 eV, further confirming that the MoO_2_ could be formed on the MoSe_2_ in the calcination process [35]. Another peak located at 532.0 eV could be attributed to the trace water adsorbed.

**Figure 5:**
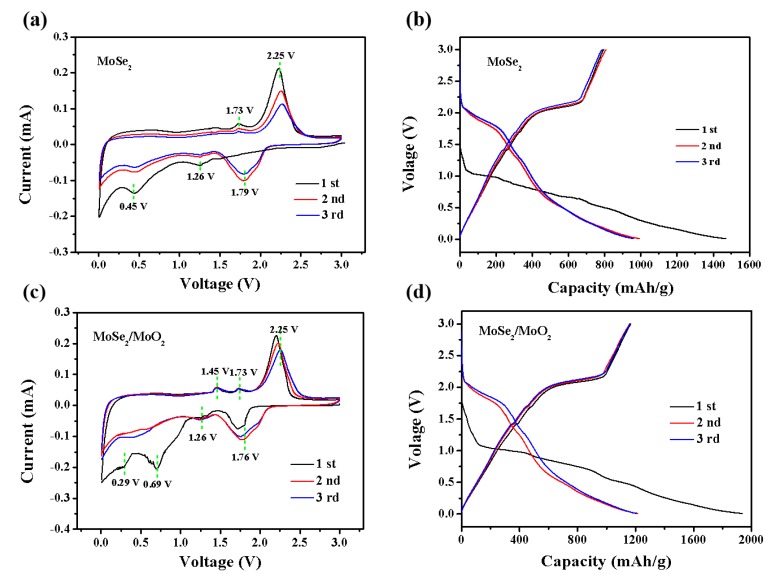
CV curves of (**a**) MoSe_2_ and (**c**) MoSe_2_/MoO_2_ anodes in the potential range 0.01–3.0 V at a scan rate of 0.1 mV s^−1^, The first three voltage-capacity profiles of (**b**) MoSe_2_ and (**d**) MoSe_2_/MoO_2_ anode at a current density of 0.1 A g^−1^.

Figure 5a shows CV curves for the initial three cycles of the MoSe_2_ anode at 0.1 mV s^−1^. The cathodic peaks observed at 1.26 V during the first cycle could be attributed to the insertion of Li^+^ into MoSe_2_ nanosheets to form Li_x_MoSe_2_ [36]. The cathodic peak at 0.45 V is assigned to the further conversion from Li_x_MoSe_2_ to Mo and Li_2_Se, as well as the formation of solid electrolyte interphase (SEI) layer [37]. Hereafter, the cathodic peak appeared at 1.79 V is equivalent to the conversion of Se to Li_2_Se and the association of Li with Mo. In the anodic process, the peak at 1.73 and 2.25 V correspond to the conversion of Li_2_Se to Se and Mo to MoSe_2_ [38]. It is noteworthy that the CV curves of the second and third cycles have large deviations in shape, indicating that the stability and reversibility of the MoSe_2_ anode are poor.

Figure 5b shows the galvanostatic discharge-charge profiles of MoSe_2_ anode at 0.1 A g^−1^. The MoSe_2_ anode delivers an initial discharge and charge capacity of 1473 mAh g^−1^ and 794 mAh g^−1^, corresponding to an initial coulombic efficiency (CE) as low as 53.9%. In the second and third cycle, 80.9% and 83.0% efficiency can be observed, respectively. These values were much lower than those of the MoSe_2_/MoO_2_ anode.

The CV curves of the MoSe_2_/MoO_2_ anode are shown in Figure 5c. The cathodic peaks at 1.26 V and 1.76 V and the anodic peak at 2.25 V are similar to what observed in the CV curves of MoSe_2_.In addition, the cathodic peak at 0.29 V represents the decomposition of LixMoSe_2_ to Li_2_Se and Mo in the first cycle and shifts to 0.45V in subsequent cycles. The formation of an SEI layer happens at around 0.69 eV [39]. The cathodic peaks at 1.50–2.00 V and the anodic peaks at 1.25–2.00 V could be attributed to the phase transition between the monoclinic and orthorhombic phases in the partially lithiated LixMoO_2_ [40]. The peaks in the subsequent second to third cycles are almost overlapped at the same voltage, which indicates excellent chemical and structural stability of the MoSe_2_/MoO_2_ anode caused by the synergy in the MoSe_2_/MoO_2_ composite.

The first three galvanostatic discharge-charge profiles of MoSe_2_/MoO_2_ anode at 0.1 A g^−1^ are indicated in Figure 5d, where an initial discharge and charge capacity of 1926 mAh g^−1^ and 1174 mAh g^−1^ can be achieved. The corresponding low CE is about 60.9%, attributing to the formation of SEI film, which consumes some of the original lithium [41,42]. In the subsequent two cycles, the CE is about 96.3% and 97.1%. The capacity loss from the 1st to 2nd cycle could be ascribed to the irreversible reactions during the discharge/charge processes, such as the formation of SEI film. It indicates that MoSe_2_/MoO_2_ anode possess highly reversible specific capacities and cyclic stability. In addition, according to the results of XPS, the atomic contents of Mo, Se and O elements are 32.63%, 52.28%, and 15.09%, respectively. The results show that MoSe_2_ and MoO_2_ account for 78% and 22% of the composite. Therefore, it is calculated based on theoretical specific capacity of MoSe_2_ (422 mAh g^−1^) and MoO_2_ (838 mAh g^−1^) that the theoretical capacity of MoSe_2_/MoO_2_ anode is about 514 mAh g^−1^ lower than practical capacity of 1926 mAh g^−1^, which is attributed to the synergistic effect of MoSe_2_ and MoO_2_.

**Figure 6:**
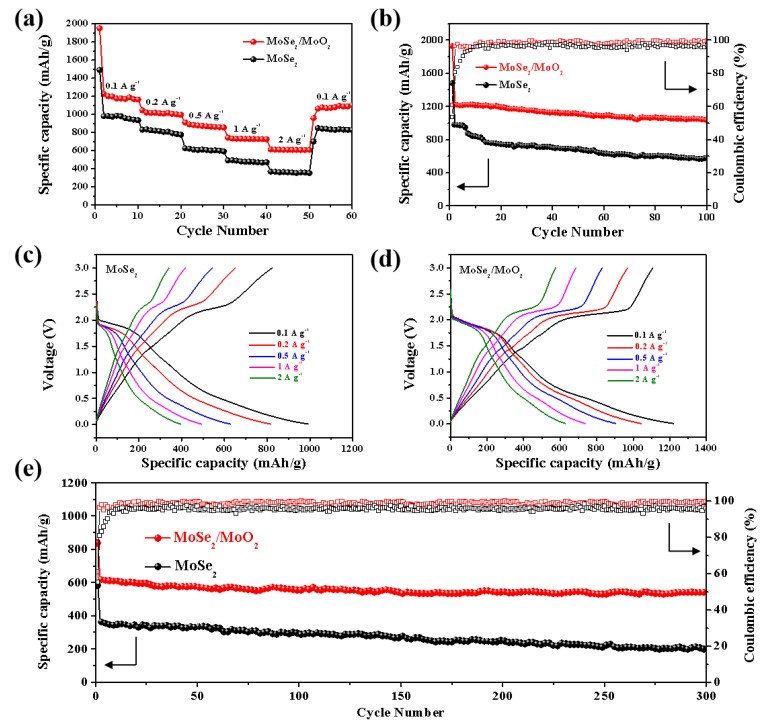
(**a**) Rate capacity at different current densities (increased from 0.1 A g^−1^ to 2 A g^−1^) of the MoSe_2_ and MoSe_2_/MoO_2_ anode; (**b**) Cycling performance of MoSe_2_ and MoSe_2_/MoO_2_ anode at 0.1 A g^−1^; (**c**–**d**) Charge-discharge curves of MoSe_2_ and MoSe_2_/MoO_2_ anode at different current densities; (**e**) Long-term cycling performance of the MoSe_2_ and MoSe_2_/MoO_2_ anode at a rate of 2 A g^−1^.

The rate capability of the two anodes is compared in Figure 6a. MoSe_2_/MoO_2_ anode delivers the specific capacities of 1214, 1045, 903, 741, and 612 mAh g^−1^ at 0.1, 0.2, 0.5, 1, and 2 A g^−1^, respectively. In contrast, the MoSe_2_ anode only shows very low reversible capacities of 497 and 339 mAh g^−1^ at 1 and 2 A g^−1^, respectively. When the current density is taken back to 0.1 A g^−1^, it is noted that the capacity retention of the MoSe_2_/MoO_2_ anode reaches as high as 80.1%, indicating excellent rate capability of this anode. The cycling stability of MoSe_2_/MoO_2_ anode is evaluated at 0.1 A g^−1^. As shown in Figure 6b, MoSe_2_/MoO_2_ anode exhibits a high specific capacity of 1042 mAh g^−1^ after 100 cycles, and the CE is close to 100%, indicating good cycling stability. For comparison, MoSe_2_ anode only delivers a specific capacity of 581 mAh g^−1^ after 100 cycles.

The charge-discharge curves of MoSe_2_ and MoSe_2_/MoO_2_ anode at different current densities after 1st cycle in the range of 0.01–3 V are shown in Figure 6c–d. At corresponding density, MoSe_2_/MoO_2_ anode shows a higher discharge specific capacity than MoSe_2_ anode, and the charge and discharge platform is more obvious, even at 2 A g^−1^, the shape of the charge and discharge platform is still very complete. The excellent long-term cycling behavior of MoSe_2_/MoO_2_ anode at 2 A g^−1^ is also exhibited in Figure 6e. The MoSe_2_/MoO_2_ anode preserves a high discharge capacity of 547 mAh g^−1^ with the CE close to 100% after 300 cycles. On the other hand, the MoSe_2_ anode exhibits a discharge capacity of 202 mAh g^−1^, much lower than MoSe_2_/MoO_2_ anode. The excellent electrochemical properties of MoSe_2_/MoO_2_ anode can be attributed to the promotion of Li^+^ transport by MoSe_2_ and protection of lamellar structure of MoSe_2_ by MoO_2_.

**Figure 7:**
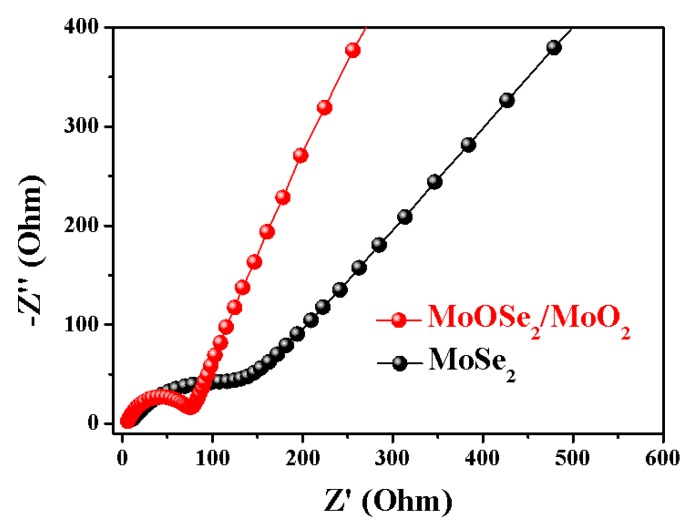
Nyquist plots for MoSe_2_ and MoSe_2_/MoO_2_ anode.

The EIS of both anodes is shown in Figure 7. All EIS plots consist of a semi-circle in the medium-high frequency region and a straight line in the low-frequency region, representing the charge transfer process and typical Warburg behavior. From the results, the optimized MoSe_2_/MoO_2_ anode shows a smaller semi-circle diameter than the MoSe_2_ anode, indicating its lowest charge transfer resistance (77 Ω). In the low frequencies, as-prepared MoSe_2_/MoO_2_ composite has the largest slope demonstrating the best lithium diffusion efficiency, which means that the electrochemical reaction becomes easier than MoSe_2_ anode [43]. It is contributed to the nanoscale combination effect promotes the diffusion and transfer of lithium, which is a key factor in obtaining high-performance LIBs.

## 4. Conclusions

MoSe_2_/MoO_2_ composite is obtained by a simple method with calcinating. It can alleviate huge volume variation of composite, promote the transmission of Li^+^ and provide a high interfacial area of electrode/electrolyte, which demonstrates excellent electrochemical properties. For example, the MoSe_2_/MoO_2_ composite exhibits a high capacity of 547 mAh g^−1^ after 300 cycles at 2 A g^−1^. The simple and effective synthesis of this composite has great potential for the application of Selenium-based materials in LIBs.
